# Clinical improvement of necrobiosis lipoidica with topical ruxolitinib

**DOI:** 10.1016/j.jdcr.2026.08.055

**Published:** 2026-09-08

**Authors:** Madeline Guy, Dennis Vidmar, Janet Wieselthier, Elma Baron, Elaine Kunzler

**Affiliations:** a College of Medicine, Northeast Ohio Medical University, Rootstown, Ohio; b Dermatology Service, Department of Veterans Affairs Louis Stokes Medical Center, Cleveland, Ohio; c Department of Dermatology, Case Western Reserve University School of Medicine, Cleveland, Ohio

**Keywords:** JAK inhibitor, Janus kinase inhibitor, necrobiosis lipoidica, ruxolitinib

## Background

Necrobiosis lipoidica (NL) is a chronic granulomatous dermatitis characterized by yellow-red-brown papules, nodules, and plaques often involving pretibial areas.^1^ NL lesions may develop atrophy, ulceration, and telangiectasias secondary to collagen degradation.^1^^,^^2^ NL is classically associated with diabetes mellitus but may also occur in association with sarcoidosis, hyperlipidemia, inflammatory bowel disease, autoimmune thyroiditis, rheumatoid arthritis, monoclonal gammopathy, or idiopathically.^2^ It has a predilection for women, affecting females 2 to 3 times more often than men with a typical onset in the third to fourth decade of life.^1^^,^^2^ To date, no Food and Drug Administration-approved therapy exists.^1^

Granulomatous inflammatory disorders such as NL occur via macrophage activation and proliferation, being notoriously difficult to treat.^3^ Recent studies have identified upregulation of inflammatory cytokines, such as interferon gamma (IFN-γ), contributing to constitutive activation of the janus kinase-signal transducer and activator of transcription (JAK-STAT) signaling pathway in granulomatous diseases.^3^^,^^4^ Targeting this pathway with JAK inhibition provides a promising therapeutic approach to NL, supported by clinical success in granulomatous diseases such as sarcoidosis.^3^ This report describes a case of long-standing necrobiosis lipoidica demonstrating clinical improvement following treatment with topical ruxolitinib 1.5% cream.

## Case report

A 51-year-old woman with diabetes mellitus and hypothyroidism presented to our dermatology clinic in 2014 for evaluation of a several-year history of a dermatitis involving the bilateral lower anterior legs. Her dermatological history was notable for Schamberg disease, plantar hyperkeratosis, and hand dermatitis. On physical exam, there were pink-brown papules in a linear distribution, some with overlying ulcerations distributed on the bilateral anterior lower legs. The lesions were painless and nonpruritic. Laboratory evaluation demonstrated a hemoglobin A1c of 7.5%. A 4 mm punch biopsy revealed focal necrotizing granulomatous dermatitis and panniculitis with neutrophils and vasculitis.

Over subsequent years, her dermatitis continued to progress on the bilateral lower extremities and she was described to have well-circumscribed 1-10 cm pink and yellow plaques with telangiectasias and episodic ulcerations suspicious for necrobiosis lipoidica (Fig 1, *A*). Multiple therapeutic approaches were attempted without durable benefit including topical clobetasol 0.05% cream, triamcinolone 0.1% ointment, urea 20% cream, tacrolimus 0.1% ointment, narrowband ultraviolet B phototherapy, and photodynamic therapy.

**Fig 1:**
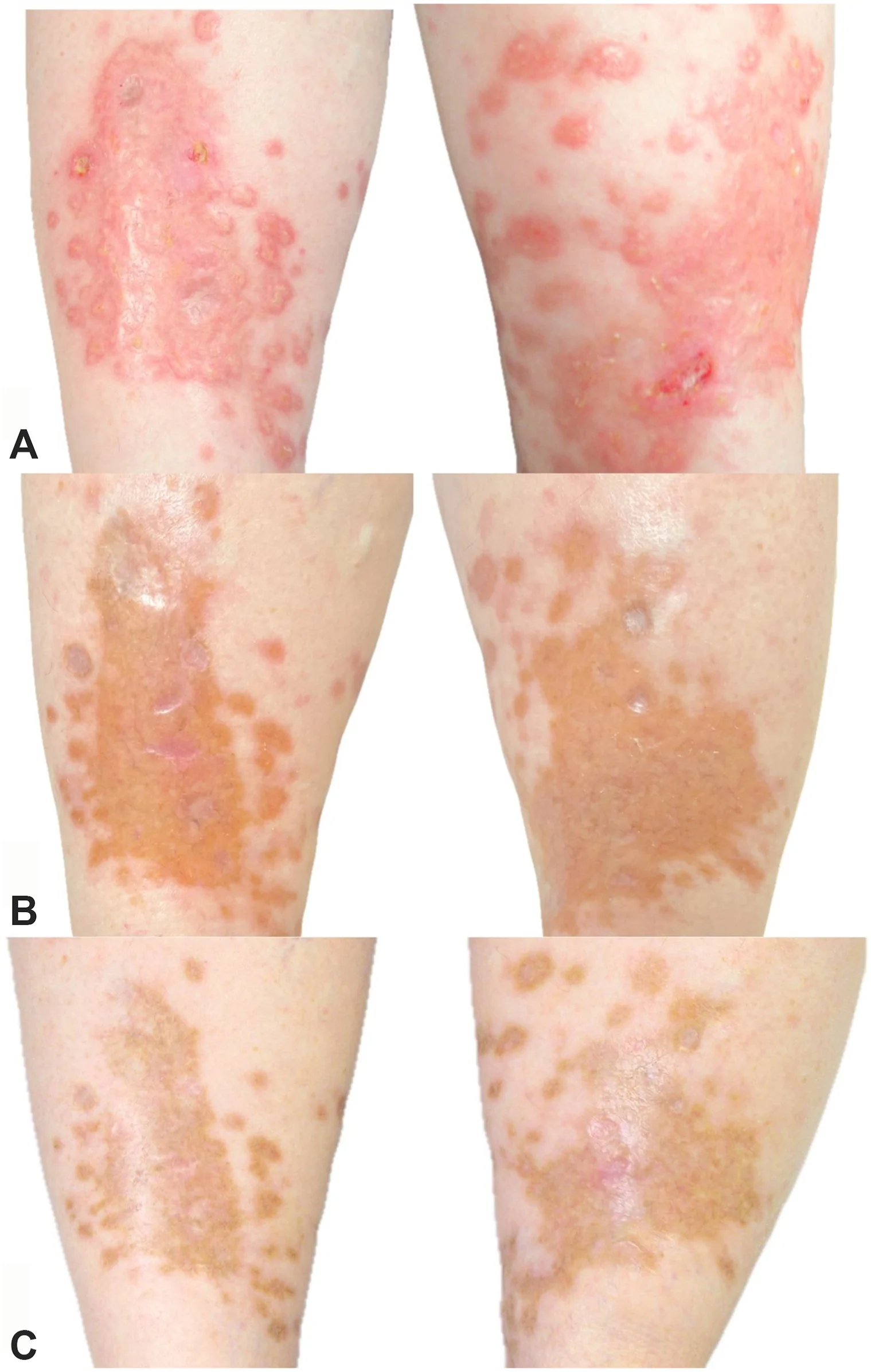
**A,** 2022: Bilateral anterior lower extremities demonstrating well circumscribed 1-10 cm pink and yellow plaques with telangiectasias and episodic ulcerations prior to initiation of ruxolitinib therapy. **B,** March 2024: Necrobiosis lipoidica progression prior to ruxolitinib therapy with persistent induration and intermittent ulceration despite therapeutic attempts with topical corticosteroids, calcineurin inhibitors, phototherapy, and urea cream. **C,** January 2025: Eight-month follow-up after initiation of topical ruxolitinib showing reduced erythema and induration, with no active ulceration or new lesion formation.

In 2022, repeat biopsy confirmed the diagnosis of necrobiosis lipoidica with histopathologic features of parakeratosis with neutrophils in the stratum corneum, diminished granular cell layer, and mild irregular acanthosis of the epidermis (Fig 2). The dermis demonstrated a superficial and deep histiocytic infiltrate palisaded with interstitial histiocytes and multinucleated giant cells, along with a mild perivascular lymphocytic infiltrate. Elastophagocytosis was present and colloidal iron staining was negative for dermal mucin. Topical tacrolimus 0.1% ointment twice daily and at-home narrowband phototherapy were reinitiated in addition to oral pentoxifylline 400 mg three times daily along with intralesional triamcinolone 10 mg/mL.

**Fig 2:**
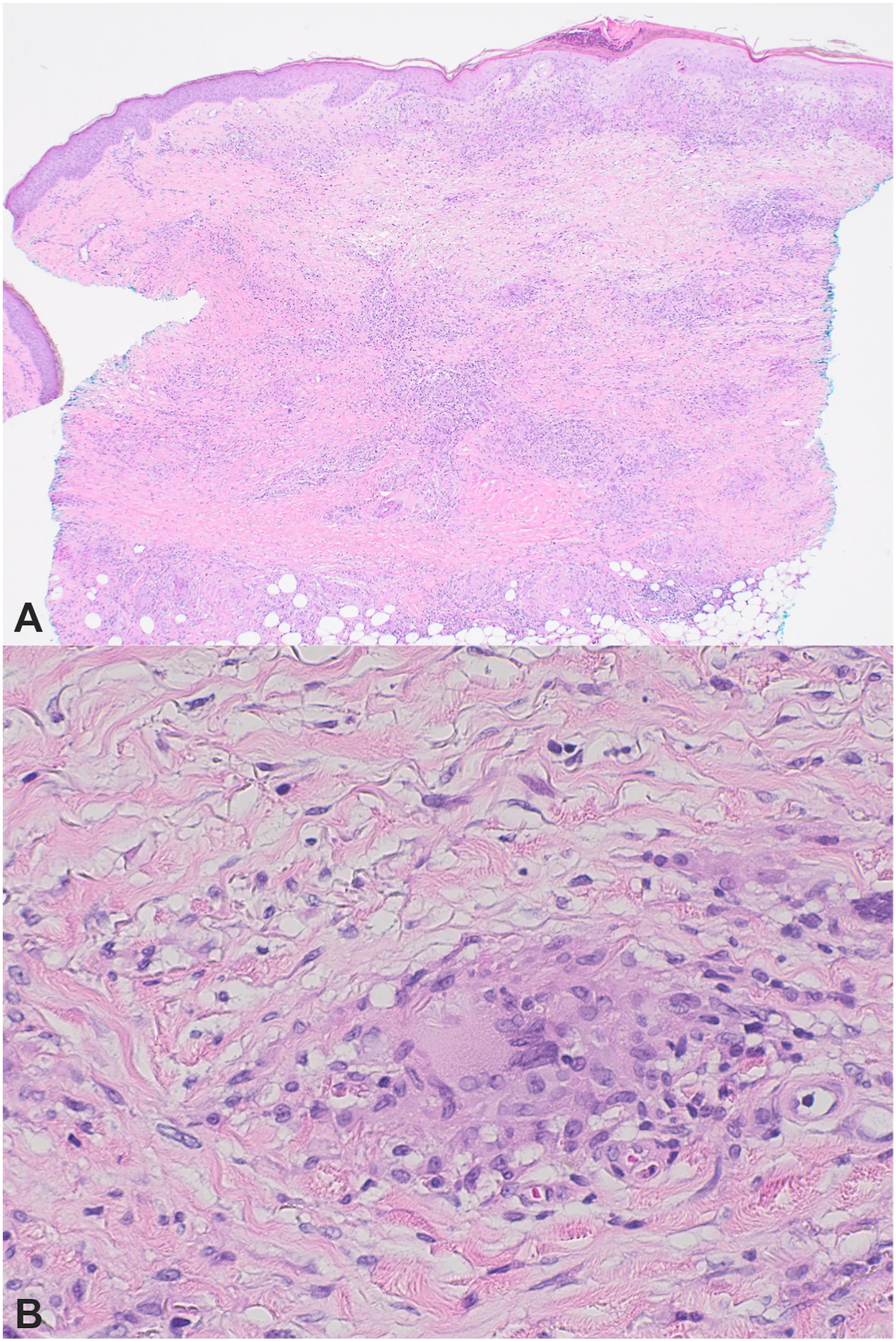
**A,** Hematoxylin and eosin stain, ×40: superficial and deep palisaded granulomatous infiltrate within the dermis. **B,** Hematoxylin and eosin stain, ×400: histiocytic infiltrate and multinucleated giant cells with a perivascular lymphocytic infiltrate surrounding collagen degeneration.

Despite these interventions, her lesions demonstrated persistent induration and intermittent ulceration, unresolved after 2 years (Fig 1, *B*). In May 2024, topical ruxolitinib 1.5% cream bid was added to her treatment regimen. After 2 months, she reported decreased pain with reepithelialization of ulcerations. At her 8 month follow up, she was noted to have continued gradual improvement with reduced erythema and induration and no active ulcerations (Fig 1, *C*). At 14 and 17 month follow ups she demonstrated sustained reduction in induration, erythema, and hyperpigmentation with persistent absence of lesions or ulcers and transient pruritus. Twenty months after ruxolitinib initiation, her exam was notable for poorly defined golden-brown atrophic plaques with minimal erythema and persistent absence of active ulceration and induration. She reported her condition to be remarkably stable and continues topical ruxolitinib 1.5% cream bid and hydrophilic cream for barrier therapy.

## Discussion

Necrobiosis lipoidica is a chronic inflammatory skin condition associated with painful ulcerations and long-term morbidity.^1^ Ulcerations pose increased risk of secondary infection and malignant transformation, requiring effective treatment and long-term monitoring.^5^ Current therapeutic modalities include topical or intralesional steroids, tumor necrosis factor alpha inhibitors, calcineurin inhibitors, biologics, photochemotherapy, pentoxifylline, interleukin 17 inhibitors, fumaric acid esters, photodynamic therapy, or surgery which have provided varied results.^4^ Therapeutic challenges may arise from limited clarity regarding disease pathogenesis, reinforcing the need for an understanding of pathophysiology.^3^

In this case, the patient’s NL was refractory to various treatment modalities for more than a decade. The rapid and sustained clinical response observed with topical ruxolitinib suggests involvement of JAK-STAT signaling in the pathogenesis of NL, with constitutive pathway activation contributing to chronic granulomatous inflammation.^4^ This is supported by prior reports of clinical remission of NL with topical JAK-STAT inhibitor therapy.^5^^,^^6^ Our patient’s response is consistent with molecular network analyses identifying IFN-γ driven JAK-STAT activation as a driver of NL and JAK inhibition as a validated therapeutic intervention.^7^

In addition, a prospective open label study published by Hwang et al identified JAK-STAT signaling and IFN-γ involvement in the pathogenesis of NL, noting significant clinical improvement, without serious adverse effects with topical ruxolitinib usage.^4^ Inhibition of this pathway utilizing ruxolitinib topical therapy offers a potential therapeutic approach to NL as it interrupts the otherwise continuous inflammatory process contributing to the morbidity of this chronic disease.

## Conflicts of interest

None disclosed.
